# Weaning from venous-arterial extracorporeal membrane oxygenation: The hemodynamic and clinical aspects of flow challenge test

**DOI:** 10.3389/fmed.2022.989197

**Published:** 2022-09-08

**Authors:** Jing-chao Luo, Yi-jie Zhang, Jun-yi Hou, Ming-hao Luo, Kai Liu, Guo-wei Tu, Zhe Luo

**Affiliations:** ^1^Department of Critical Care Medicine, Zhongshan Hospital, Fudan University, Shanghai, China; ^2^Shanghai Medical College, Fudan University, Shanghai, China; ^3^Department of Critical Care Medicine, Xiamen Branch, Zhongshan Hospital, Fudan University, Xiamen, China; ^4^Shanghai Key Lab of Pulmonary Inflammation and Injury, Shanghai, China

**Keywords:** flow challenge test, weaning evaluation, V-A ECMO, cardiogenic shock, cardiac function reserve

## Abstract

The cardiac function reserve is crucial for the successful weaning of V-A ECMO. During the V-A ECMO weaning phase, the gradual reduction in pump flow converts the blood flow originally driven by the pump to native cardiac output and also transforms afterload (caused by retrograde flow) into ventricular preload, thus introducing a “flow challenge” to the native heart. In this perspective, we propose to use this flow challenge as a test to simulate the preload-to-afterload conversion to assess cardiac functional reserve quantitatively. With this short article we offer the hemodynamic and clinical aspects regarding the flow challenge test.

## Introduction

Venous-arterial extracorporeal membrane oxygenation (V-A ECMO) is an important circulatory support to rescue patients with refractory cardiogenic shock (1, 2). However, the use of V-A ECMO can also lead to various complications (3, 4), such as infection, hemorrhage, lung injury and skeletal muscle atrophy (5). Besides, prolonged V-A ECMO support was also associated with higher mortality (6), therefore, early weaning should be considered to maximize its benefits. Before removing V-A ECMO, an accurate evaluation of the recovery of cardiac function is of crucial importance. On one hand, some patients, who failed to meet institutional criteria for weaning consistently, might have sufficient cardiac function reserve to tolerate the increased cardiac load after removal of V-A ECMO. This “unnecessary” V-A ECMO support exposed them to increased risk of subsequent complications. On the other hand, nearly one third of deceased patients had once been weaned from ECMO (7). Of note, one of the major contributors for the death after weaning was heart failure (5).

Previously, studies have proposed several cardiac systolic function parameters to predict the successful weaning of V-A ECMO, such as aortic velocity-time integral (VTI), left and right ventricular ejection fraction, lateral mitral annulus peak systolic velocity and pulse pressure (8, 9). However, these parameters may not be good predictors for successful V-A ECMO weaning, as they fail to directly reflect the native heart’s ability to cope with drastic hemodynamic changes after weaning. A more essential indicator to evaluate cardiac function reserve, that is, the ability to transfer excess blood volume (originally delivered by V-A ECMO) into the native cardiac output (CO) to maintain adequate systemic perfusion is necessary.

### Downgrading venous-arterial extracorporeal membrane oxygenation flow brings a hemodynamic challenge to the native heart

With V-A ECMO support, the global blood flow is contributed by both the V-A ECMO device and the native heart. Thus, blood flow is distributed in two systems. Peripheral V-A ECMO draws blood from the right atrium, which is collected from the tissue by the venous return system, *via* a centrifugal pump (which generates a negative pressure), delivers it to a membrane oxygenator, and then returns it to the femoral artery. The residual blood volume in the right atrium goes through the left heart and is ejected into the systemic circulation (10).

**FIGURE 1:**
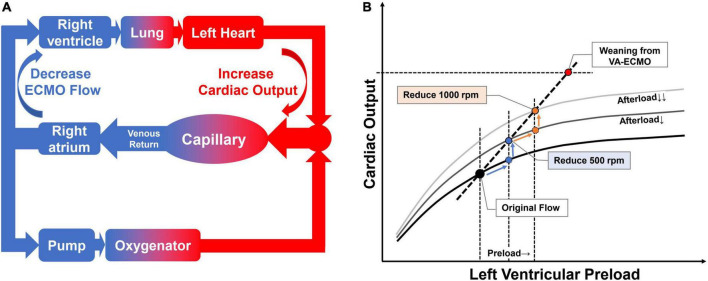
Effect of flow challenge test (FCT) on blood flow re-distribution and ventricular performance. Panel **(A)** the effect of reduced pump rotation speed on native cardiac output. When we reduce the V-A ECMO flow, the excess blood goes to the native heart and is then ejected into the systemic circulation. Panel **(B)** the flow challenge test shifts the cardiac operating points to the upper right on Frank-Starling curve. By stepping down the rotation speed of the V-A ECMO pump, the operating point shifts to the right along the Frank-Starling curve. In addition, the Frank-Starling curve itself shifts upward as afterload is reduced due to a decrease in retrograde blood flow. The combined effect is a shift of the heart’s operating point to the upper right.

The “flow challenge” is a process in which the downgrading of V-A ECMO flow is carried out to evaluate if the native heart can cope with the increased burden. During the V-A ECMO weaning phase, the speed of pump rotation is gradually reduced (8). This process of downgrading the device flow decreases the *trans*-pump pressure (suction power) leading to lower blood flow through the pump, increases the blood volume reaching the left heart (Figure 1A) (10), and therefore posing a remarkable but reversible hemodynamic challenge, which could be potentially used to evaluate the cardiac function reserve.

### From fluid challenge test to flow challenge test

The assessment of fluid responsiveness in patients with V-A ECMO could not only help to optimize the preload but also guide the decision to wean from ECMO support. In the TEMPLE study (11), a change of preload was induced either by the Trendelenburg maneuver or fluid challenge, while the pump was maintained at the same rotation speed. Ventilation support, sedation and vasopressors remained unchanged as well. This study has demonstrated that an increase in VTI of at least 10%, induced by the Trendelenburg maneuver is reliable in predicting fluid responsiveness in patients with V-A ECMO while keeping the pump flow unchanged.

Adjusting the flow of V-A ECMO centrifugal pump is a routine to simulate different loading conditions to assess the performance of ventricles during the weaning phase (8). Different from fluid challenge, reducing V-A ECMO flow (flow challenge) will increase preload but decrease afterload (12). In other words, it is a conversion of afterload to preload. Patient’s functional cardiac reserve, which was used to overcome afterload, is now converted to cope with the increased preload. Therefore, it has two main effects: (1) blood flow reaching the left heart will increase accordingly and move the operating point along the Frank-Starling curve to the right; (2) decreased retrograde device flow reduces afterload and thus shifts the Frank-Starling curve upward (Figure 1B). The combined effect is a shift of the heart’s operating point to the upper right. This maneuver could be called flow challenge test (FCT).

### Using flow challenge test maneuver to depict the relationship between venous-arterial extracorporeal membrane oxygenation flow and native CO

**FIGURE 2:**
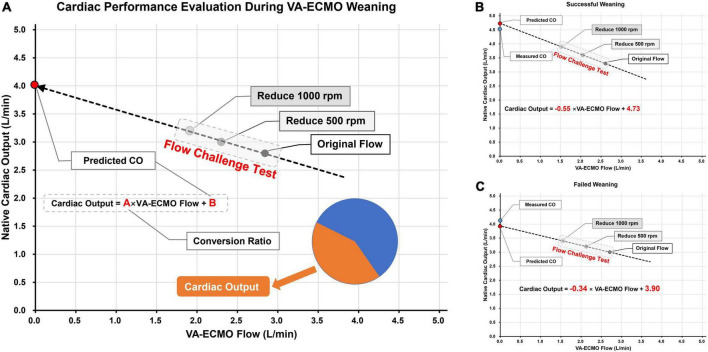
Schematic diagram of using flow challenge test (FCT) to predict cardiac output (CO) performance after V-A ECMO weaning. Panel **(A)** the picture on the left is the schematic diagram of FCT; The pump speed was reduced on two stages, each time by 500 rpm. CO were measured at the corresponding three points and the actual ECMO flow rates were also recorded. The CO after weaning was then predicted by linear regression and the conversion ratio between ECMO blood flow and CO was calculated. Panels **(B,C)** two cases of successful and failed weaning, respectively. The predicted and actual values of CO are very close to each other. The weaning successful patient shown higher predicted CO and conversion ratio than the weaning failure patient.

The speed of the pump could be gradually decreased when decisions are made to start the weaning evaluation process. Two types of flow reduction protocol are widely used in clinical studies, a proportional approach where a certain percentage of rotating speed is withdrawn (13), or an equidistant approach where a certain number of rotating speed is deducted (14). In our center, reducing the pump rotation speed involves two steps, each at 500 rpm (0.5 L/min equivalently). After reducing the pump rotation speed, the V-A ECMO flow will decrease, which in turn increases the blood flow back to the native heart. On a scatter plot, using V-A ECMO flow as the horizontal axis and the native CO as the vertical axis, the regression line corresponding to the three points can thus be obtained (Figure 2A). The intercept of the vertical axis represents the predicted immediate CO after ECMO weaning, and the slope indicates the **conversion ratio** between reduced ECMO flow and increased CO.

### Prediction of CO after weaning by flow challenge test maneuver

Here we present two detailed cases underwent FCT before V-A ECMO weaning. Both patients received V-A ECMO support for cardiogenic shock. Cardiac function was gradually recovered in both patients, and V-A ECMO blood flow rate was reduced to around 2.5 L/min. During the weaning process, transthoracic echocardiography was used to measure VTI (at the level of the left ventricular outflow tract) to calculate the CO. After pump speed reduction, V-A ECMO flow and CO were recorded after a 10-min stabilization. In the first case (Figure 2B), during the FCT process, the V-A ECMO flow decreased from 2.61 to 2.05 and 1.53 L/min while CO increased from 3.3 to 3.6 to 3.9 L/min, sequentially. The linear regression formula is Cardiac Output = −0.55 × V-A ECMO flow + 4.73, with a vertical axis intercept of 4.73 (the predicted CO value immediately after V-A ECMO weaning), which is extremely close to the actual measurement (4.5 L/min). In the second case (Figure 2C), the FCT also induced a well-fitted regression line (Cardiac Output = −0.34 × V-A ECMO flow + 3.90). The predicted and measured values of CO were also very close (3.9 vs. 4.1 L/min).

### Assessment of cardiac functional reserve by flow challenge test maneuver

In the aforementioned two cases, changes in the central venous pressure (CVP) were observed after weaning from V-A ECMO. During the FCT maneuver, CVP almost remained the same. We assumed it was because reflecting the subtle changes in cardiac preload under right atrial V-A ECMO drainage was difficult. After removal of V-A ECMO, the CVP of the two cases increased from 13 to 15 mmHg, and 14 to 19 mmHg, respectively, suggesting that more blood was retained in the venous and right heart system after weaning from V-A ECMO. This also means that the blood flow that was originally supplied by the device was not fully compensated by the native heart. Instead, the recovering heart could only convert part of the ECMO flow into native CO, while the unconverted part was transferred into stressed volume, achieving a new balance.

The concept of conversion ratio was then investigated. As we have previously defined, the conversion ratio indicates the ability of the heart to convert the reduced V-A ECMO flow into native CO. In the first case, the conversion ratio was 0.55, which meant every 1 L/min reduction in V-A ECMO flow could lead to 0.55 L/min increment in CO. However, in the second case, the conversion ratio was only 0.34. The two cases also had different outcomes, with the first case weaned successfully while the second one failed. Therefore, the conversion ratio has the potential to become a quantitative parameter for assessing cardiac functional reserve.

### Enriching evidence for clinical application

Before formal clinical application can be made, several studies should be performed to provide more evidence. (1) *in vitro* simulation tests: digital cardiovascular system models (15) or the mock circulatory loops (16) could be used to simulate the conversion ratios corresponding to FCT performed with different cardiac function reserves. (2) Animal models: In the animal V-A ECMO model, we can place a variety of pressure and flow transducers and perform detailed transesophageal echocardiographic monitoring to assess whether the cardiovascular response, when performing FCT under different cardiac functions, is consistent with that predicted by the *in vitro* model. (3) Clinical observational study: In a larger cohort of population, evaluate the associations between conversion ratio or predicted CO and cardiac functional parameters or clinical outcomes, and then calculate their best cutoff values for weaning. (4) Interventional study: Use the conversion ratio and predicted CO at FCT to guide weaning and assess whether this tool could improve prognosis.

## Summary

Flow challenge test is easy to implement during the V-A ECMO weaning phase and can provide quantifiable measures in guiding V-A ECMO management. During FCT, afterload is converted into preload. This process can introduce a hemodynamic challenge and be used to predict the CO value after weaning, which is an important determinant of systemic oxygen delivery and tissue perfusion. In addition, the proposed “conversion ratio” as a parameter of cardiac functional reserve measured during the process of FCT, has the potential to be used as a dynamic parameter to improve the accuracy of conventional static ones in the field of hemodynamic monitoring. The dynamic process of FCT could allow us to make more rational predictions about the instant cardiac consequences of V-A ECMO weaning.

## Data availability statement

The raw data supporting the conclusions of this article will be made available by the authors, without undue reservation.

## Ethics statement

Written informed consent was obtained from the individual(s) for the publication of any potentially identifiable images or data included in this manuscript.

## Author contributions

J-CL, Y-JZ, and J-YH: development, authoring, editing, and final version approval of the manuscript. All authors read and approved the final manuscript.

## References

[1] Rali AS Chandler J Sauer A Solomon MA Shah Z. Venoarterial extracorporeal membrane oxygenation in cardiogenic shock: Lifeline of modern day CICU. *J Intensive Care Med.* (2021) 36:290–303. 10.1177/0885066619894541 31830842

[2] Pineton de Chambrun M Brechot N Lebreton G Schmidt M Hekimian G Demondion P Venoarterial extracorporeal membrane oxygenation for refractory cardiogenic shock post-cardiac arrest. *Intensive Care Med.* (2016) 42:1999–2007. 10.1007/s00134-016-4541-y 27681706

[3] Hou D Wang H Yang F Hou X. Neurologic complications in adult post-cardiotomy cardiogenic shock patients receiving venoarterial extracorporeal membrane oxygenation: A cohort study. *Front Med.* (2021) 8:721774. 10.3389/fmed.2021.721774 34458294 PMC8385654

[4] Roumy A Liaudet L Rusca M Marcucci C Kirsch M. Pulmonary complications associated with veno-arterial extra-corporeal membrane oxygenation: A comprehensive review. *Crit Care.* (2020) 24:212. 10.1186/s13054-020-02937-z 32393326 PMC7216520

[5] Luo JC Zheng WH Meng C Zhou H Xu Y Tu GW Levosimendan to facilitate weaning from cardiorespiratory support in critically Ill patients: A meta-analysis. *Front Med.* (2021) 8:741108. 10.3389/fmed.2021.741108 34712681 PMC8546177

[6] Lee HH Kim HC Ahn CM Lee SJ Hong SJ Yang JH Association between timing of extracorporeal membrane oxygenation and clinical outcomes in refractory cardiogenic shock. *JACC Cardiovasc Interv.* (2021) 14:1109–19. 10.1016/j.jcin.2021.03.048 34016408

[7] Smith M Vukomanovic A Brodie D Thiagarajan R Rycus P Buscher H. Duration of veno-arterial extracorporeal life support (VA ECMO) and outcome: An analysis of the extracorporeal life support organization (ELSO) registry. *Crit Care.* (2017) 21:45. 10.1186/s13054-017-1633-1 28264702 PMC5339999

[8] Ortuno S Delmas C Diehl JL Bailleul C Lancelot A Naili M Weaning from veno-arterial extra-corporeal membrane oxygenation: Which strategy to use? *Ann Cardiothorac Surg.* (2019) 8:E1–8. 10.21037/acs.2018.08.05 30854330 PMC6379199

[9] Su Y Liu K Zheng JL Li X Zhu DM Zhang Y Hemodynamic monitoring in patients with venoarterial extracorporeal membrane oxygenation. *Ann Transl Med.* (2020) 8:792. 10.21037/atm.2020.03.186 32647717 PMC7333156

[10] Le Gall A Follin A Cholley B Mantz J Aissaoui N Pirracchio R. Veno-arterial-ECMO in the intensive care unit: From technical aspects to clinical practice. *Anaesth Crit Care Pain Med.* (2018) 37:259–68. 10.1016/j.accpm.2017.08.007 29033360

[11] Luo JC Su Y Dong LL Hou JY Li X Zhang Y Trendelenburg maneuver predicts fluid responsiveness in patients on veno-arterial extracorporeal membrane oxygenation. *Ann Intensive Care.* (2021) 11:16. 10.1186/s13613-021-00811-x 33496906 PMC7838230

[12] Aissaoui N Guerot E Combes A Delouche A Chastre J Leprince P Two-dimensional strain rate and Doppler tissue myocardial velocities: Analysis by echocardiography of hemodynamic and functional changes of the failed left ventricle during different degrees of extracorporeal life support. *J Am Soc Echocardiogr.* (2012) 25:632–40. 10.1016/j.echo.2012.02.009 22421027

[13] Aissaoui N Luyt CE Leprince P Trouillet JL Leger P Pavie A Predictors of successful extracorporeal membrane oxygenation (ECMO) weaning after assistance for refractory cardiogenic shock. *Intensive Care Med.* (2011) 37:1738–45. 10.1007/s00134-011-2358-2 21965097

[14] Cavarocchi NC Pitcher HT Yang Q Karbowski P Miessau J Hastings HM Weaning of extracorporeal membrane oxygenation using continuous hemodynamic transesophageal echocardiography. *J Thorac Cardiovasc Surg.* (2013) 146:1474–9. 10.1016/j.jtcvs.2013.06.055 23993027

[15] Huang H Yang M Zang W Wu S Pang Y. *In vitro* identification of four-element windkessel models based on iterated unscented Kalman filter. *IEEE Trans Biomed Eng.* (2011) 58:2672–80. 10.1109/TBME.2011.2161477 21859593

[16] Tozzi P Maertens A Emery J Joseph S Kirsch M Avellan F. An original valveless artificial heart providing pulsatile flow tested in mock circulatory loops. *Int J Artif Organs.* (2017) 40:683–9. 10.5301/ijao.5000634 28862717

